# Symbol Digit Modalities Test Variant in a Smartphone App for Persons With Multiple Sclerosis: Validation Study

**DOI:** 10.2196/18160

**Published:** 2020-10-05

**Authors:** Pim van Oirschot, Marco Heerings, Karine Wendrich, Bram den Teuling, Marijn B Martens, Peter J Jongen

**Affiliations:** 1 Orikami Digital Health Products Nijmegen Netherlands; 2 Dutch National MS Foundation Rotterdam Netherlands; 3 Radboud University Medical Center Nijmegen Netherlands; 4 Faculty of Science, Institute for Science in Society Radboud University Nijmegen Netherlands; 5 Drug Target ID Nijmegen Netherlands; 6 NeuroDrug Research BV Nijmegen Netherlands; 7 Department of Community & Occupational Medicine University Medical Centre Groningen Groningen Netherlands; 8 MS4 Research Institute Nijmegen Netherlands

**Keywords:** relapsing-remitting multiple sclerosis, cognition, processing speed, mobile phone

## Abstract

**Background:**

The decline of cognitive processing speed (CPS) is a common dysfunction in persons with multiple sclerosis (MS). The Symbol Digit Modalities Test (SDMT) is widely used to formally quantify CPS. We implemented a variant of the SDMT in MS sherpa, a smartphone app for persons with MS.

**Objective:**

The aim of this study was to investigate the construct validity and test-retest reliability of the MS sherpa smartphone variant of the SDMT (sSDMT).

**Methods:**

We performed a validation study with 25 persons with relapsing-remitting MS and 79 healthy control (HC) subjects. In the HC group, 21 subjects were matched to the persons with MS with regard to age, gender, and education and they followed the same assessment schedule as the persons with MS (the “HC matched” group) and 58 subjects had a less intense assessment schedule to determine reference values (the “HC normative” group). Intraclass correlation coefficients (ICCs) were determined between the paper-and-pencil SDMT and its smartphone variant (sSDMT) on 2 occasions, 4 weeks apart. Other ICCs were determined for test-retest reliability, which were derived from 10 smartphone tests per study participant, with 3 days in between each test. Seven study participants with MS were interviewed regarding their experiences with the sSDMT.

**Results:**

The SDMT scores were on average 12.06% higher than the sSDMT scores, with a standard deviation of 10.68%. An ICC of 0.838 was found for the construct validity of the sSDMT in the combined analysis of persons with MS and HC subjects. Average ICCs for test-retest reliability of the sSDMT for persons with MS, the HC matched group, and the HC normative group were 0.874, 0.857, and 0.867, respectively. The practice effect was significant between the first and the second test of the persons with MS and the HC matched group and trivial for all other test-retests. The interviewed study participants expressed a positive attitude toward the sSDMT, but they also discussed the importance of adapting a smartphone cognition test in accordance with the needs of the individual persons with MS.

**Conclusions:**

The high correlation between sSDMT and the conventional SDMT scores indicates a very good construct validity. Similarly, high correlations underpin a very good test-retest reliability of the sSDMT. We conclude that the sSDMT has the potential to be used as a tool to monitor CPS in persons with MS, both in clinical studies and in clinical practice.

## Introduction

### Background

Multiple sclerosis (MS) is a chronic disease in which the body’s immune system mistakenly attacks the isolating sheath (myelin) that surrounds the nerve fibers in the central nervous system. MS may affect the brain, the spinal cord, and the optic nerves, and disrupt the information flow across the affected nerves. This may cause a variety of symptoms, including loss of vision (optic neuritis), muscle weakness, sensory symptoms, cognitive dysfunction, altered coordination, and fatigue. For most persons with MS, sudden relapses contribute to the unpredictability of the disease, which makes it difficult to devise a treatment plan specific to an individual patient.

The prevalence of cognitive dysfunction in persons with MS is estimated to be between 40% and 70%, depending on the research setting [1-4]. This can include dysfunction in visuospatial processing, cognitive processing speed (CPS), working memory, executive functioning, verbal and visual learning, as well as episodic memory. CPS decline is among the first cognitive signs and the most commonly observed cognitive deficit in persons with MS. Importantly, CPS decline has a significant impact on the quality of life [3,5]. CPS is usually assessed by measuring the amount of information processed in a unit of time or the time needed to process a given amount of information.

The Paced Auditory Serial Addition Test (PASAT) [6] and the Symbol Digit Modalities Test (SDMT) [7] are the most widely used tests to formally quantify CPS in MS, and they focus on auditory CPS and visuospatial CPS, respectively [8-10]. Both are included in the Rao Brief Repeatable Battery of Neuropsychological tests, amongst tests for learning and executive function. Sometimes, the Brief Repeatable Battery of Neuropsychological tests is administered by clinicians during check-ups [2,11]. Both tests are part of the Minimal Assessment of Cognitive Function [12]. Notably, the more compact Brief International Cognitive Assessment for MS (BICAMS) test battery prefers SDMT over PASAT [13]. The SDMT score has a strong correlation with the Expanded Disability Status Scale (EDSS) score [14]—a stronger correlation than the PASAT scores [15,16]—as well as with abnormalities seen in magnetic resonance images such as in brain lesion volume, cerebral atrophy, diffusion tensor indices of normal appearing brain tissue, and retinal nerve fiber layer thickness [17]. Although both the PASAT and SDMT are highly sensitive in detecting cognitive impairment, the SDMT has higher acceptability among patients, is easier to administer, and has slightly higher sensitivity than PASAT [13,15]. Moreover, findings suggest that the SDMT more accurately reflects the qualitative nature of self-reported cognitive impairment and should perhaps replace the PASAT as part of the MS Functional Composite (MSFC) [18]. Finally, the SDMT has been found to be relatively resistant to practice effects and is therefore useful for serial testing and screening [19].

Although the SDMT has been proven to be clinically relevant, it is not regularly used in standard care. This is partly because patients often do not complain about cognitive changes during consultation with a health care professional. It is also because of the time factor and administrative burden, despite the relatively short duration of one assessment, that is, 90 seconds. Paper-and-pencil variants of the SDMT have been existing for quite some time [20,21], but digital variants have been developed only in recent years for computers [18], tablets [22,23], or smartphones [24,25]. It is believed that e-versions will make it easier to monitor CPS. However, to our knowledge, no scientific papers about the validity of the smartphone variants of the SMDT have been published to date.

MS sherpa (Orikami Digital Health Products) is a smartphone app intended to support the monitoring of persons with MS, in order to give patients and their health care professionals personalized insight into the presence and progress of MS-related symptoms and signs. MS sherpa contains a smartphone variant of the SDMT (sSDMT). This sSDMT works as follows: a large black symbol present in the middle of the screen has to be matched to the correct digit. Matching can be done at the bottom of the screen. Once an answer is given, a new symbol appears. The sSDMT score is the number of correct answers in this 90-second test. A small stopwatch counting down is shown above the numbers at the bottom of the screen. The key is shown at the top of the screen. The same key is used during 1 assessment, but it varies between assessments; the symbols are randomly matched to digits in the key each time a test is done. These symbols are different from those in the original SDMT since these are protected by copyrights.

Note that contrary to the original SDMT, in sSDMT, one cannot look ahead as to which symbol is to be matched next. However, also in the original SDMT, skipping a symbol in the presented sequence is not allowed. There are 2 other aspects in which the sSDMT differs from the SDMT. First, in the original SDMT, the first 26 items are selected from the first 6 symbols in the key. In the sSDMT, symbols are selected from the full key during the whole assessment. This was also the case in the first version of other alternative versions of the SDMT [5]. Second, instead of using the first 10 “practice” items that can be matched with guidance in the original SDMT, it is possible to practice the sSDMT by pressing the corresponding button on the instruction screen. In a practice test, symbols are again randomly matched to digits in the key, which is therefore different from the key in the “real” sSDMT. The rationale for not matching exactly the “practice” phase to the original SDMT was that home monitoring can be done frequently and practicing before every test should not be mandatory, from a usability perspective.

### Objective

We aimed to study the construct validity and test-retest reliability of the sSDMT that is implemented in MS sherpa as an assessment for CPS and present our findings in this paper, along with respondents’ experiences with the sSDMT. The validation of the sSDMT was part of the MS Self study. The MS Self study was a validation study during which study participants performed self-monitoring assessments during 4 weeks with a precursor of MS sherpa, which was called the “Mijn Kwik” app and a Fitbit Charge 2 wearable. Besides investigating the validity of the sSDMT, another research objective of the MS Self study was to investigate first the experiences with digital self-monitoring through smartphone apps and activity trackers of persons with MS by interviewing 7 study participants with MS. Furthermore, the study aimed to validate a smartphone variant of the 2-minute walking test (s2MWT) and a smartphone walking balance test (sWBT). In particular, we investigated if the outcomes of the smartphone tests were in agreement with the outcomes of corresponding clinical tests, namely, SDMT, the 2-minute walking test (2MWT), and the Timed Up and Go test (TUG). The other results from the interviews as well as a description of the methodology have recently been published [26]. Separate research papers on the s2MWT, the sWBT, and the Fitbit data are in preparation. A poster with the preliminary results of the MS Self study [27] can be found on the MS sherpa website [28]. This poster also contains an image of the sSDMT.

## Methods

### Study Design

This study was performed in 25 persons with relapsing-remitting MS and 2 groups of healthy control (HC) subjects (n=79). The HC subjects in the first control group (HC matched, n=21) were matched to the persons with MS with regard to age, gender, and education. Five education categories were defined: “secondary general education,” “senior secondary general education and preuniversity education,” “secondary vocational education,” “higher professional education,” and “scientific education”. The second control group (HC normative, n=58) was set up to determine the normal distribution for the smartphone test results.

The inclusion criteria were as follows: (1) willing to participate and capable of doing all the tasks mentioned in the protocol, (2) able and willing to use own smartphone, which should be an iPhone 5 or a newer Apple device, or a phone with an Android operating system version 6 or higher, and (3) aged between 20 years and 50 years. For persons with MS, the following additional inclusion criteria were set: (1) diagnosis of relapsing-remitting MS for more than one year and (2) having an EDSS score between 1.5 and 6.5. A maximal EDSS score of 6.5 is needed to perform the 2MWT. A minimal EDSS score of 1.5 was chosen to find a difference in the 2MWT results for persons with MS and HC subjects.

**Figure 1:**
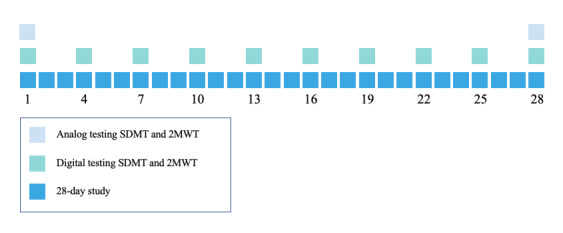
Overview of the study design and assessment scheme. SDMT: Symbol Digit Modalities Test; 2MWT: 2-minute walking test.

On the first and the last day of the study, persons with MS and the HC matched group came to the premises of the Dutch National MS Foundation (Nationaal MS Fonds [NMSF]) to perform a paper-and-pencil SDMT, followed by a simultaneously performed 2MWT and a s2MWT in the open air. Later that day, and at most 24 hours later, they also performed the sSDMT in their home environment. During the 4-week follow-up, these study participants performed the s2MWT and sSDMT tests at home, once every 3 days, that is, 10 times in total. A schematic overview of the study design is given in Figure 1. The EDSS was administered to every person with MS by MH on the first day of the study.

The HC subjects in the HC normative group were instructed to do the s2MWT and the sSDMT 3 times in total, with 1 week in between the assessments. From these tests and from the 10 home assessments of the other study participants, the test-retest reliability of the sSDMT was determined. The combined test results “SDMT-sSDMT” for the persons with MS and the HC matched group were used to determine the construct validity of the sSDMT. This combination will hereafter be referred to as the validation assessment. Note that there are 2 validation assessments per study participant: one in the beginning and one at the end of the study.

### Recruitment

The persons with MS and HC matched group were recruited via the NMSF (Rotterdam, the Netherlands). The HC normative group was recruited via the social network of the app manufacturer. 

### Ethical Approval and Informed Consent

This study was approved by the medical ethical committee METC Brabant (Tilburg, the Netherlands) under protocol number NL61291.028.17. All study participants agreed to the privacy statement of the Mijn Kwik app prior to first use. Persons with MS and the HC matched group signed an informed consent letter on paper.

### Data Collection

Data were collected between May 2017 and May 2018. The ages of the HC subjects in the HC matched group were matched as closely as possible to that of the persons with MS, which led to at most 2 years difference for 86% (18/21) of the pairs. Approximately 90% (19/21) of the pairs matched in gender. An exact match in education category was obtained for 29% (6/21) of the pairs.

### Data Analysis

In the analyses performed in this study, the SDMT score on the first day of the study was compared with the first sSDMT score and the SDMT score on the last day of the study was compared with the last sSDMT score. In the data cleaning process of the sSDMT data, the home assessments in which the study participants had a score below 20 were removed because these were outliers. It was assumed that the users were distracted during these tests. A score below 20 occurred in less than 1% (3/423) of all the sSDMTs that were done.

### Statistical Analysis

Following the study protocol, the significance level α was set to 5%. Two-sided *t* tests were conducted to compare the SDMT score with the sSDMT score. For the smartphone tests, the median test score of each study participant was taken in the *t* test analysis. Internal consistency was evaluated and quantified using Cronbach α, in which an α value larger than .7 was defined to be acceptable [29,30]. Test-retest reliability was determined by calculating the intraclass correlation coefficient (ICC) between measurements at different times. ICCs smaller than 0.59 were considered to be “moderate,” ICCs between 0.60 and 0.79 were considered “good,” and ICCs above 0.80 were considered “very good” [31,32]. The effect size Cohen *d* was determined to investigate the practice effect. A Cohen *d* below 0.20 was seen as trivial [33].

To investigate the construct validity of the sSDMT, we calculated the (1) ICC between the SDMT and the sSDMT and the (2) Pearson correlation coefficient between the SDMT and the sSDMT (the distributions were checked for normality by applying Shapiro-Wilk tests).

For these statistical tests, we used the same acceptance criteria as for the test-retest reliability: values smaller than 0.59 were considered to be “moderate,” values between 0.60 and 0.79 were considered “good,” and values above 0.80 were considered “very good.”

We calculated different ICCs for the construct validity and the test-retest reliability, following the McGraw and Wong [34] convention. Although the “model” selection (two-way mixed effects) and the “type” selection (single rater/measurement) of both ICCs are the same, the “definition” (consistency or absolute agreement) is different [35]. ICCs of type “ICC(3,1)” were calculated to compare the SDMT and the sSDMT. These ICCs have definition “consistency,” since these 2 test scores were not expected to exactly match. ICCs of type “ICC(A,1)” were calculated to determine test-retest reliability. These ICCs have definition “absolute agreement,” since the test scores per individual were not expected to change within the 1-month follow-up of the study.

We checked if the distributions of the number of correct answers on the first sSDMT that was done for the 3 populations in this study were normally distributed via Shapiro-Wilk tests and if the 2 groups of the HC subjects had the same underlying distribution via a 2-sample Kolmogorov-Smirnov test. Finally, we did a two-sided *t* test to confirm the difference between persons with MS and HC subjects.

## Results

### Participant Demographics

The mean, median, and standard deviation in the ages for the various groups are listed in Table 1, as well as the gender, the number of participants in each education category (for the HC normative group, no education information was collected), and the mean, median, and standard deviation in the EDSS score (persons with MS).

**Table 1:** Participant demographics.

Characteristics		Persons with MS^a^, n=25	HC^b^ matched, n=21	HC normative, n=58
**Age (years)**				
	Mean (SD)	40 (8)	37 (8)	34 (8)
	Median	43	36	32
**Gender**				
	Female, n (%)	23 (92)	17 (81)	29 (50)
	Male, n (%)	2 (8)	4 (19)	29 (50)
**Education**				
	Secondary general education, n (%)	1 (4)	3 (14)	—^c^
	Senior secondary general education and preuniversity education, n (%)	3 (12)	3 (14)	—
	Secondary vocational education, n (%)	8 (32)	3 (14)	—
	Higher professional education, n (%)	9 (36)	6 (29)	—
	Scientific education, n (%)	4 (16)	6 (29)	—
**EDSS^d^ score**				
	Mean (SD)	3.1 (1.4)	N/A^e^	N/A
	Median	3.0	N/A	N/A

^a^MS: multiple sclerosis.

^b^HC: healthy control.

^c^Not available.

^d^EDSS: Expanded Disability Status Scale.

^e^N/A: not applicable.

Besides these 104 study participants, 2 more persons were recruited, but 1 person with MS dropped out of the study because she was experiencing a relapse and 1 HC dropped out because she did not like wearing Fitbit.

### Distinctions Between Persons with MS and HC Subjects

**Figure 2:**
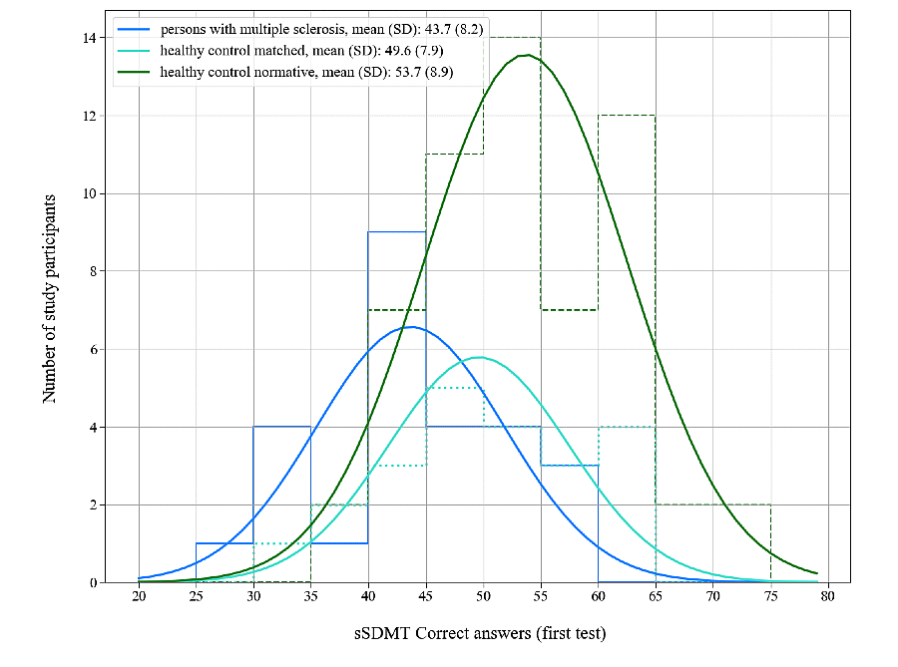
Distributions in the number of correct answers on the first test done with the sSDMT for the 3 groups in this study. The thin solid line shows the distribution for persons with multiple sclerosis, the dotted line shows the distribution for the healthy control matched group, and the dashed line shows the distribution for the healthy control normative group. The thick solid lines represent Gaussian fits to the distributions, of which the means (SD) are shown in the legend. sSDMT: smartphone variant of Symbol Digit Modalities Test.

Figure 2 shows the number of correct answers for the first sSDMT in the persons with MS and in the 2 control groups. The number of correct answers was normally distributed for all 3 groups as calculated by the Shapiro-Wilk tests (*P*=.49, *P*=.29, and *P*=.37 for persons with MS, the HC matched group, and the HC normative group, respectively). The 2 groups of HC subjects have the same underlying distribution as confirmed using a 2-sample Kolmogorov-Smirnov test (Kolmogorov-Smirnov statistic=0.26, *P*=.20). Independent 2-sample *t* tests between persons with MS versus the HC matched group and persons with MS versus the HC normative group confirmed that the sSDMT can distinguish between persons with MS and HC subjects at the group level (*P*=.02 and *P*<.001, respectively).

### Construct Validity

Two variants of the conventional SDMT were used in the validation assessments: the original paper version of the SDMT [7] (abbreviated as “SDMT”) and a paper version of the sSDMT (a variant of the SDMT, hereafter referred to as “vSDMT”). Since the SDMT is copyright protected, the vSDMT was printed in the study protocol. This was misinterpreted by some members of the research team (PvO and MH) as an extra variant of the SDMT to be investigated and was randomly assigned to more than half of the study participants. Different variants could therefore be used in the 2 validation assessments of a given person. Contrary to the regular instructions for an SDMT, study participants did not get 10 “practice” items to be matched with guidance in this study neither for the vSDMT nor for the SDMT. In total, 37 SDMTs and 55 vSDMTs were conducted. In this section, we present the comparison between the SDMT and the sSDMT (raw data in Multimedia Appendix 1). The raw data of the validation assessments with vSDMTs are listed in Multimedia Appendix 2.

The construct validity was determined from 37 validation assessments with the SDMT. One of these assessments was the first validation assessments of a dropout. The vSDMT score of the first validation assessments of the other dropout is included in the Multimedia Appendix 2. However, including these assessments did not increase the total number of validation assessments since 1 HC subject did 2 invalid SDMTs. This person did not match the symbols with the digits in the order of appearance on the sheet of paper, but instead started matching all symbols of a specific kind first, both on the first and on the last day of the study.

**Figure 3:**
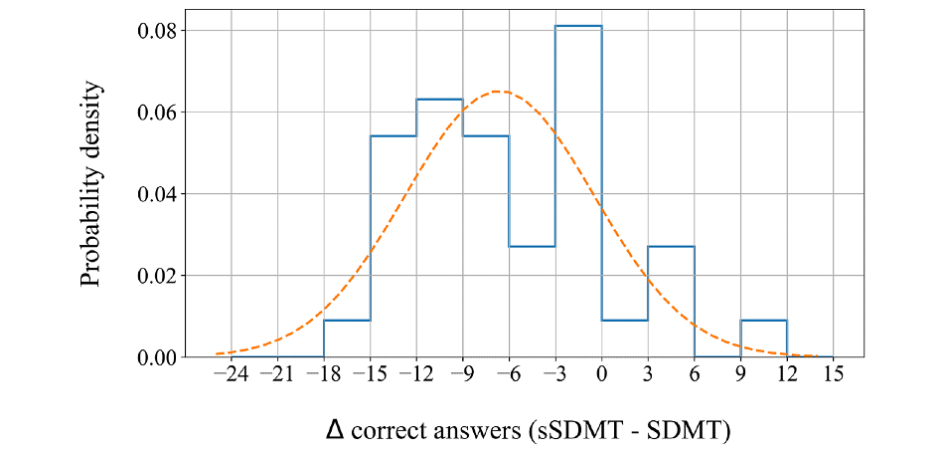
Distribution of the differences between the number of correct answers on the sSDMT and the SDMT. The dashed line represents a normal distribution. sSDMT: smartphone variant of Symbol Digit Modalities Test; SDMT: Symbol Digit Modalities Test.

**Figure 4:**
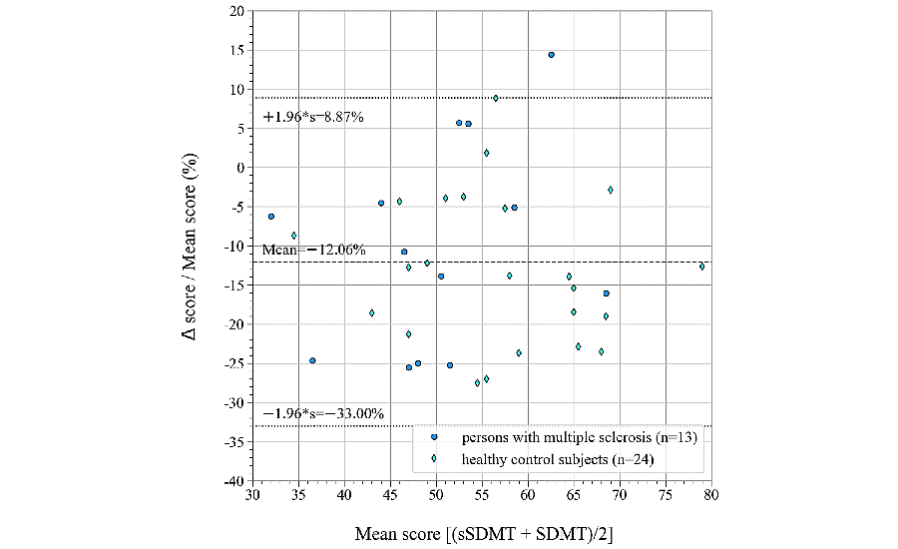
Bland-Altman plot of differences between the number of correct answers on the sSDMT and SDMT, expressed as percentages of the mean value (∆/mean) versus the mean of the 2 measurements (raw data in Multimedia Appendix 1). The dashed line shows the mean percentage difference, and the dotted lines show the 95% confidence interval. sSDMT: smartphone variant of Symbol Digit Modalities Test; SDMT: Symbol Digit Modalities Test.

It was found that, on average, 6.62 less correct answers were given on the sSDMT than on the SDMT (Figure 3). A Shapiro-Wilk test on the distribution of the differences accepted normality (*P=*.15). This allows us to determine the 95% CI, at 1.96*s around the mean difference, with SD=6.13 correct answers, which is visualized in the Bland-Altman plot (Figure 4). In this plot, the difference between the 2 tests is shown as a percentage of the mean of the 2 test outcomes on the vertical axis, and the mean is shown on the horizontal axis. The number of correct answers on the sSDMT is, on average, 12.06% (6.62/54.80) lower than the number of correct answers on the SDMT (*P*<.001). The 1.96*s above and below this average results in a difference of 8.87% more and 33.00% less correct answers on the sSDMT than on the SDMT, respectively.

**Figure 5:**
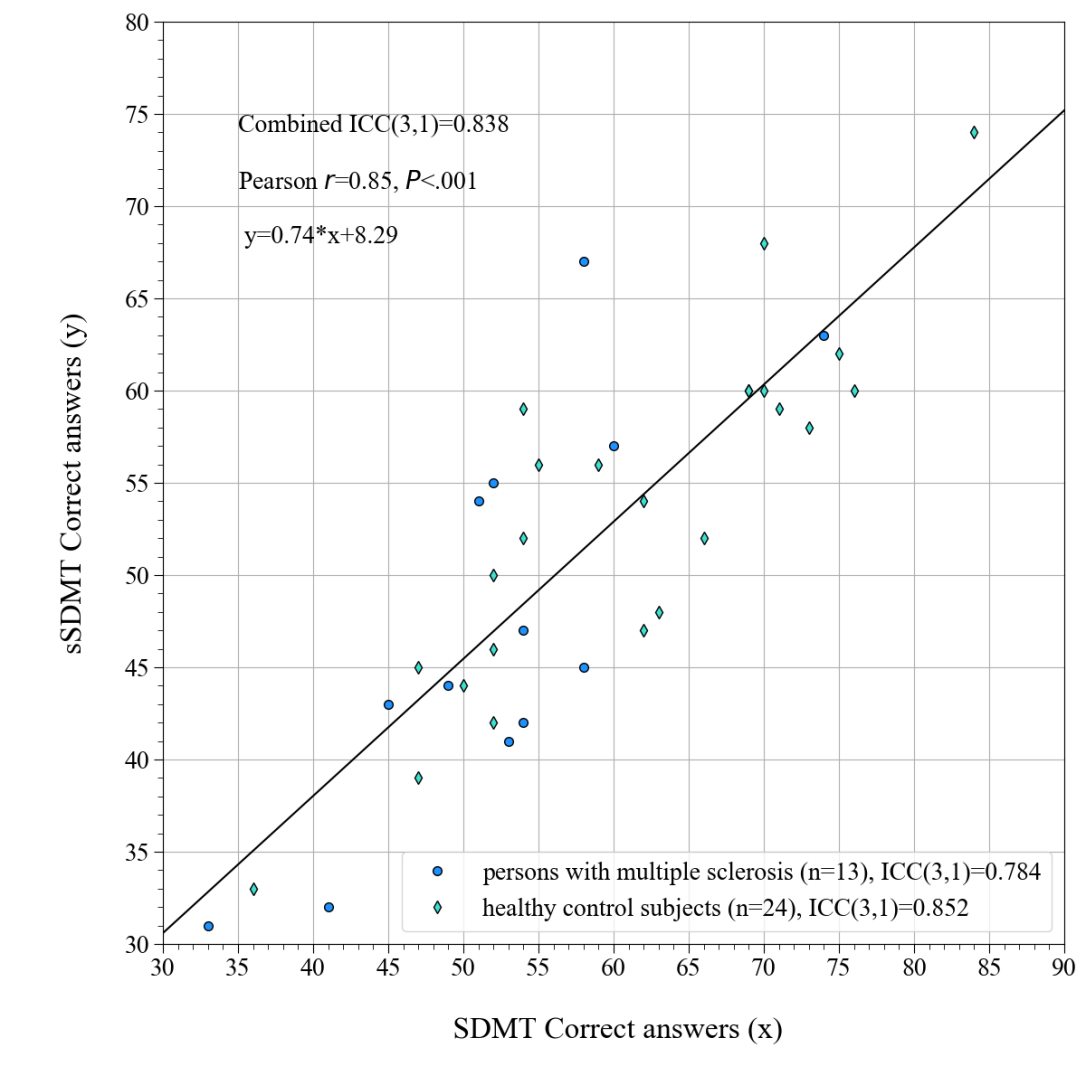
Scatter plot showing the ICCs(3,1) and the correlation (Pearson r, upper left corner) between the number of correct answers on the SDMT (horizontal axis, “x”) and the sSDMT (vertical axis, “y”). A linear fit through the data points is visualized as a black solid line, for which the formula is also given in the upper left corner. ICC: intraclass correlation coefficient; sSDMT: smartphone variant of Symbol Digit Modalities Test; SDMT: Symbol Digit Modalities Test.

When investigating the agreement between the SDMT and the sSDMT using a *t* test, as was planned in the study protocol, a very small *P* value was obtained. A dependent *t* test for paired samples yielded a test statistic of 6.49 (*P*<.001). Since this was below the significance level of .05, we rejected the null hypothesis of identical average scores. In Figure 5, the number of correct answers on the sSDMT is plotted against the number of correct answers on the SDMT. ICCs(3,1) were determined for persons with MS and HC subjects separately and for the combined dataset, which are shown in the top left corner of Figure 5. The Pearson correlation coefficient and the formula corresponding to the linear fit through the data are also shown in the top left corner of Figure 5.

**Figure 6:**
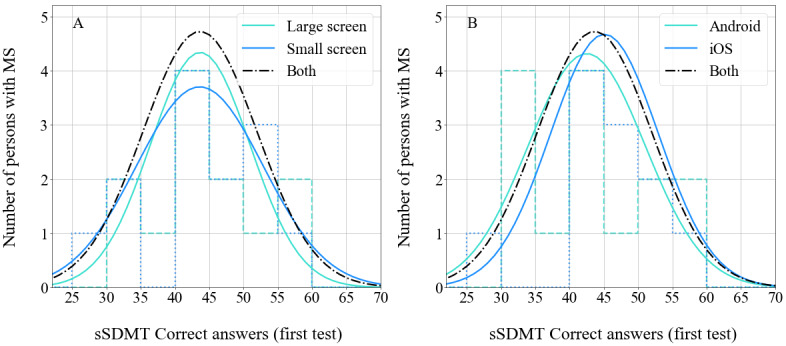
A: Distribution of the number of correct answers on the first sSDMT of 13 persons with multiple sclerosis who used a smartphone with a large screen size (dashed line) and 13 persons with multiple sclerosis who used a smartphone with a small screen size (dotted line). The thick solid lines represent Gaussian fits to the distributions. B: Distribution of the number of correct answers on the first sSDMT of 11 persons with multiple sclerosis who used a smartphone with an iPhone operating system (iOS) (dotted line) and 15 persons with multiple sclerosis who used a smartphone with an Android operating system (dashed line). The thick solid lines represent Gaussian fits to the distributions. In both panels, the dot-dashed line represents a Gaussian fit to the full distribution (both categories). MS: multiple sclerosis; sSDMT: smartphone variant of Symbol Digit Modalities Test.

For the persons with MS, we checked if the number of correct answers on the first sSDMT was dependent on the screen size of their smartphones or their operating systems. The resulting distributions are respectively shown in the left and right panels of Figure 6. We took the total number of pixels as an estimate for screen size. The screens with less than 2 million pixels were considered small, while those with 2 million pixels or more were considered large. The number of correct answers was normally distributed for all 4 categories as calculated by the Shapiro-Wilk tests (*P*=.54, *P*=.48, *P*=.16, and *P*=.30 for a small screen, large screen, iPhone operating system, or Android operating system, respectively). Since we investigated the score on the first sSDMT, we also included the test results of the persons with MS who dropped out of the study. We cannot confirm if this dropout did not experience difficulties with perceptual motor abilities, but inclusion of this individual does not significantly affect the results.

The 2 categories of screen size as well as the 2 categories of operating systems had the same underlying distribution as confirmed using 2-sample Kolmogorov-Smirnov tests (Kolmogorov-Smirnov statistic for screen size=0.23, *P*=.83 and Kolmogorov-Smirnov statistic for operating system=0.28, *P*=.60). These distributions are plotted with a dot-dashed line in both panels of Figure 6. Similarly, independent 2-sample *t* tests between the 2 categories of screen size and between the 2 categories of operating systems confirmed that neither the screen size nor the operating system had an effect on the test score (*P*=.98 and *P*=.46, respectively).

### Test-Retest Reliability

One of the dropouts did 7 home assessments with the sSDMT. We consider this a sufficiently large number to calculate test-retest reliability; therefore, we included this dropout for the analyses.

**Figure 7:**
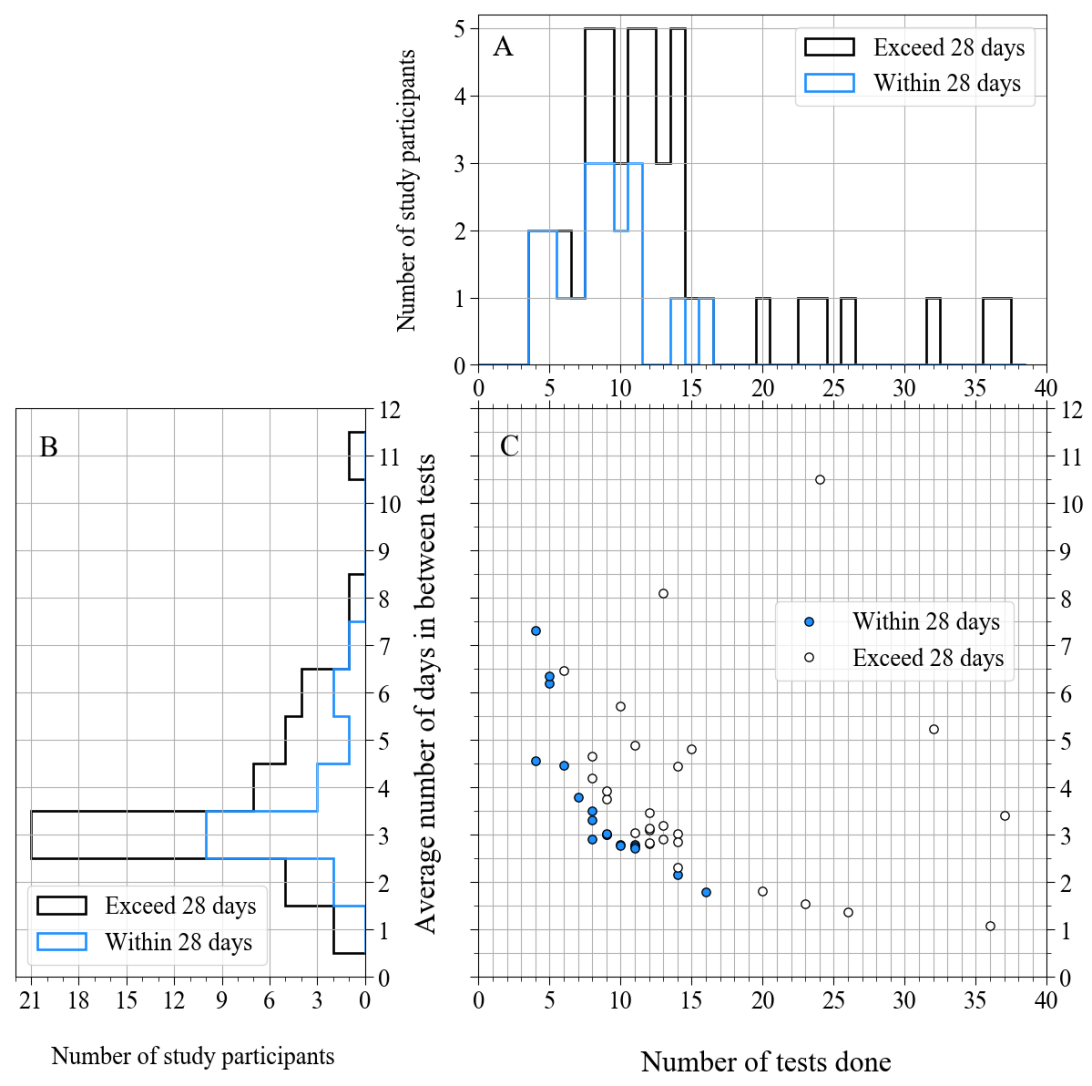
A: Histogram showing the distribution of total number of tests done. B: Histogram showing the distribution of number of days in between tests, averaged over all tests of a study participant. C: Scatter plot that shows the relation between the number of tests done (horizontal axis) and the average number of days in between tests, averaged over all tests of a study participant (vertical axis) for all study participants. Filled circles correspond to participants that finished the study within 28 days, open circles correspond to those for which the study duration exceeded 28 days. The blue lines in panels A and B correspond to these filled circles, and the black lines in panels A and B correspond to the open circles.

Figure 7A shows the distribution of the total number of home assessments done for persons with MS and HC matched group. Even though 57% (27/47) of the persons with MS and HC subjects in the matched group did more than 10 tests in total, we included only their first 10 home assessments for the test-retest reliability calculations, as planned in the study protocol. Figure 7B shows the distribution of the average number of days the persons with MS and HC subjects in the matched group left between tests, which was averaged over all tests of a study participant. This distribution peaks at 3 days, but it is different from 3 days for 55% (26/47) of the study participants. In these histograms, we also show the distributions of 60% (28/47) of the persons with MS and HC subjects in the matched group who continued to do tests after 4 weeks. The scatter plot in the lower right panel of Figure 7 (Figure 7C) shows the relation between the number of tests done and the average number of days in between tests. Study participants who did all home assessments within 28 days are plotted with filled circles, and the others with open circles.

The ICCs(A,1) (with their 95% CI), Cronbach α, and Cohen *d* values that were derived from 9 test-retests of the sSDMT for the persons with MS and the HC matched group are listed in Table 2, and the values of those derived from the 2 test-retests performed by the HC normative group are shown in Table 3. The raw data from persons with MS and the HC matched group can be found in the Multimedia Appendix 3 and that of the HC normative group in Multimedia Appendix 4. The mean values of the ICC(A,1) for persons with MS, HC matched group and the HC normative group were 0.874, 0.857, and 0.867, respectively.

**Table 2:** Test-retest reliability scores ICC(A,1), Cronbach α, and Cohen d of the sSDMT for persons with multiple sclerosis and the healthy control matched group. The numbers in the parentheses after the ICCs(A,1) indicate lower and upper boundaries of the 95% confidence interval, respectively.

Test-retest	ICC^a^(A,1) persons with MS^b^	ICC(A,1), HC^c^ subjects	Cronbach α of persons with MS	Cronbach α of HC subjects	Cohen *d* of persons with MS	Cohen *d* of HC subjects
1	0.747 (0.297-0.902)	0.735 (0.401-0.888)	.903	.875	0.477	0.384
2	0.937 (0.863-0.972)	0.859 (0.688-0.940)	.967	.926	0.062	0.153
3	0.899 (0.788-0.953)	0.885 (0.740-0.952)	.945	.943	0.030	0.160
4	0.849 (0.690-0.930)	0.797 (0.554-0.916)	.922	.889	0.160	0.169
5	0.805 (0.611-0.909)	0.820 (0.567-0.931)	.893	.895	0.138	0.000
6	0.880 (0.752-0.944)	0.913 (0.764-0.970)	.934	.952	0.041	0.072
7	0.939 (0.867-0.972)	0.837 (0.586-0.942)	.968	.908	0.059	0.095
8	0.915 (0.814-0.963)	0.961 (0.871-0.989)	.956	.978	0.094	0.000
9	0.898 (0.769-0.957)	0.904 (0.644-0.977)	.946	.946	0.107	0.098
mean	0.874 (0.717-0.945)	0.857 (0.646-0.945)	.937	.924	0.130	0.126

^a^ICC: intraclass correlation coefficient.

^b^MS: multiple sclerosis.

^b^HC: healthy control.

**Table 3:** Test-retest reliability scores ICC(A,1), Cronbach α, and Cohen d of the sSDMT for the healthy control normative group. The numbers in the parentheses after the ICCs(A,1) indicate the lower and upper boundaries of the 95% CI, respectively.

Test-retest	ICC^a^(A,1)	Cronbach α	Cohen *d*
sSDMT^b^ test-retest 1	0.877 (0.772-0.931)	.943	0.201
sSDMT test-retest 2	0.857 (0.769-0.913)	.925	0.116
mean	0.867 (0.771-0.922)	.934	0.159

^a^ICC: intraclass correlation coefficient.

^b^sSDMT: smartphone variant of Symbol Digit Modalities Test.

As was planned, we quantified the practice effect using the effect size Cohen *d*, with a value below 0.20 being trivial (Cohen, 1988). The Cohen *d* values in Table 3 show that there is a significant practice effect after the first sSDMT for all study participants. When comparing the first and second sSDMT scores of the persons with MS and HC subjects in the matched group, we found that the mean increase in the number of correct answers in the second test was 3.38 points or 6.87% compared to the first test. Toward the end of the study, the practice effect on the sSDMT completely disappeared at group level. For example, there was, on average, no difference in the scores between the seventh and the eighth sSDMT.

Study participants performed better on the SDMT on the last day of the study, on average, compared to their score on the first day of the study. The average increase between these 2 tests was 5.57%, with a standard deviation of 11.40%, as can be calculated for the 14 study participants that did the SDMT both at the first and at the last day of the study, and even differences as high as 34.92% are reached. Comparing the first and last sSDMT for these 14 study participants yielded, on average, an increase in the number of correct answers of 11.77%, with a standard deviation of 7.84%. The Cohen *d* values quantifying the practice effect on the SDMT and the sSDMT between the first and the last day of the study were found to be 0.365 and 0.908, respectively. The practice effect for the HC normative group was also investigated. The Cohen *d* values corresponding to the first and the second retest were found to be 0.201 and 0.116 respectively (see Table 3). Although a mean increase in the test score of 3.32% at group level was found between the first and the second test with a standard deviation of 7.29%, the practice effect could be considered trivial for this group on the basis of the Cohen *d* values.

### Interview Results

The 7 participants with MS who were interviewed about their experiences with the smartphone app and Fitbit activity tracker in general expressed a positive attitude regarding sSDMT. They liked doing the test, often describing it as a “game” or a “puzzle.” Moreover, 5 respondents imagined that this test could provide valuable information to health care providers about the health status of their patients, as this is relevant information about how their patients are doing. Despite this general positive attitude, respondents also expressed some remarks regarding the sSDMT. Two respondents noticed that sometimes the digits they pressed did not seem to respond. They were unsure whether this was caused by the app or their phone. Furthermore, several respondents discussed that in order to use a smartphone cognition test in their daily life, such a test should become personalized. Three respondents mentioned that every person with MS has different difficulties regarding cognition and felt that these differences could not be captured by a single test. Rather, they envisioned a smartphone app with multiple cognition tests from which persons with MS can choose from, depending on their personal cognitive issues. Another point of personalization concerned the test frequency. Four respondents expected that during the stable periods of their MS, they would feel less need to perform the sSDMT than during periods of relapse. Therefore, they desired flexibility in the sSDMT test frequency when they would use the smartphone app in their daily life.

## Discussion

### Principal Findings

We found that the sSDMT can distinguish between persons with MS and HC subjects at the group level. The test scores on the sSDMT are, on average, 12.06% lower than that on the paper-and-pencil SDMT. Although there is no exact agreement between the sSDMT and the SDMT, the 2 tests are strongly correlated, with ICC(3,1) values of 0.784 and 0.852 for persons with MS and HC subjects, respectively. The sSDMT shows very good test-retest reliability, with average ICCs of 0.874, 0.857, and 0.867 for persons with MS, the HC matched group, and the HC normative group, respectively. The practice effect was significant between the first and the second test of the persons with MS and the HC matched group and trivial for all other test-retests. A positive attitude toward the sSDMT was found during the interviews with study participants with MS. Importantly, interview respondents expressed the desire to adapt smartphone cognition tests according to the individual needs of persons with MS, with regard to both the type and frequency of testing.

### Limitations

The SDMT is a relevant tool both for screening and monitoring not only for MS but for many other clinical diseases such as Huntington disease, Parkinson disease, and stroke [36]. This study limits itself to persons with MS, and the validity of the sSDMT will still have to be demonstrated for applications outside MS. The validation and study population are limited to Dutch people and the Dutch language. We expect that the construct validity and test-retest reliability would have been equally good if this study had been done with persons with MS from various European countries, given the validity and reliability that were found in studies in populations from various countries with other digital assessment devices for CPS. The MS sherpa app now also supports an English version of the sSDMT, and a German version is planned. However, we recommend a separate validation for each country where the sSDMT will be used, for example, as done for BICAMS [37,38] because the SDMT is known to be affected by culture [39]. It was not expected that the number of correct answers on the sSDMT and the SDMT would exactly match, because the sSDMT was not designed to follow the SDMT as close as possible, and systematic differences between the tests exist. Therefore, the SDMT and sSDMT may not be used interchangeably for monitoring. The lower scores on the sSDMT compared to the SDMT are most likely caused by the fact that all items of the sSDMT are selected from the full key, whereas in the SDMT, the first 26 items are selected from the first 6 symbols in the key only.

A 10% difference between 2 SDMT scores of a patient is found to be an indication of a clinically meaningful change in the patients’ CPS [8]. This study was not designed to derive the boundary for meaningful change on the sSDMT score. Even though there is a strong correlation between the SDMT and the sSDMT, we cannot claim that a 10% difference on 2 sSDMT scores also signifies a clinically meaningful change. Moreover, we found more than 10% increase in sSDMT performance between the first and the last day of the study, on average, even though the disability of the persons with MS was not expected to change during the 4 weeks of follow-up. An explanation for this increase and for the Cohen *d* value quantifying the practice effect on the sSDMT between the first and the last day of the study of 0.908 is that 60% (28/47) of the study participants had been learning on the sSDMT every 3 days or more often, leading to accumulations of learning effects.

One might wonder how often an sSDMT could be scheduled, to keep its value as an objective instrument for measuring CPS. Benedict et al [19] showed that the SDMT has good-to-excellent reproducibility over repeated testing when used in monthly successive examinations. The HC subjects in the normative group who had a time of 1 week between tests instead of 3 days had less of a practice effect than the persons with MS and HC subjects in the matched group. Therefore, when trying to minimize the practice effect, the scheduling frequency of self-assessments should be considered for clinical practice. Our current suggestion would be to schedule the sSDMT once a month to avoid inducing large practice effects when supporting monitoring for clinical practice.

This study was not set up to cross-device validate the sSDMT, but we have tried to give some sense of the validity over different screen sizes and the main software platforms, because we believed that these 2 factors could affect the results. In our study, we found an approximately equal number of study participants using the Android or iPhone operating system platform, which gave us the opportunity to significantly show that there is no structural bias in using any of the platforms. A more structural approach to cross-device validation and more detailed analysis of the performance over different devices could help to detect validity issues in different devices.

In our study, we did not explicitly give the instructions to self-monitor under the same conditions each time as much as possible (preferably in a quiet room). We expect that such instructions would improve the test-retest reliability. The fact that the ICCs and Cronbach α values of the first test-retest of the persons with MS and the HC matched group were lower than all following 8 can likely be explained by the fact that there is, in general, a large practice effect between the first and the second time a cognitive test is done, especially with only 3 days in between the tests. This is also reflected in the Cohen *d* values, which are the highest for the first test-retest. We explain the triviality of the Cohen *d* values corresponding to the first and the second retest of the HC normative group with the larger time in between successive tests (approximately 1 week).

The fact that 60% (28/47) of the persons with MS and HC subjects in the matched group continued to do home assessments after 4 weeks might be because the end day of the study could often not be planned 4 weeks after the first day. It could also be a sign of high acceptance of the sSDMT, as expressed by the interviewed study participants. These results, together with the qualitative findings that participants liked doing the sSDMT, often describing it as a “game” or a “puzzle,” support that the sSDMT was viewed as “game-like” and confirms that the sSDMT is well-designed for the user.

Since persons with MS were asked to write down their answers on the paper-and-pencil SDMT themselves, persons with MS who had limited hand dexterity might perform lower on the paper-and-pencil SDMT than those with the same CPS without hand dexterity problems. There was no Nine Hole Peg Test scheduled in this study to investigate this possible bias. During the sSDMT, persons with MS are asked to tap the correct answer. We expect hand dexterity problems to be less of a problem for tapping on a smartphone than for writing down a number on paper. An alternative solution for this bias would be an oral sSDMT, which should then be validated against an oral SDMT. However, persons with MS might not find this a natural way of self-monitoring.

The number of study participants for which the test-retest reliability of the paper-and-pencil SDMT could be studied was relatively low, because 2 different versions of the paper-and-pencil SDMT were used. Although the reliability of the paper-and-pencil SDMT is well known in literature, it would have been interesting to study the practice effect of frequently performing our sSDMT on the paper-and-pencil SDMT in more detail.

Finally, an important limitation is the omission of the 10 mandatory practice items on the paper-and-pencil SDMT. This could have introduced a bias toward a better correlation with the sSDMT, because in the digital variant, practicing is optional, and therefore it might affect our construct validity. The construct validity should therefore ideally be reevaluated in a follow-up study in which study participants do the standard 10 practice items on the paper-and-pencil SDMT.

As mentioned in the Introduction, originally our rationale for an optional practice assessment was that practicing should not be mandatory for persons with MS that do the sSDMT repeatedly from a usability perspective. Practicing was optional but could be done unlimitedly by pressing the corresponding button on the instruction screen. However, our results have let us to reconsider this perspective and we now believe that the sSDMT nor a practice session should be accessible unlimitedly, because of the accumulative nature of the practice effect. However, when the sSDMT is scheduled only once a month, users might want to practice before each digital assessment. Therefore, we currently suggest 10 mandatory practice items before each digital assessment, which is also more similar to the paper-and-pencil SDMT, and therefore might improve the construct validity.

### Comparison With Prior Work

A fair number of computerized neuropsychological assessment devices for monitoring cognitive impairment in MS has been developed in recent years. A recent systematic review of the literature on test batteries and single tests with good evidence for reliability and validity yielded 44 CPS tests, of which all computerized tests based on SDMT correlated with the conventional SDMT, with correlation coefficients ranging from 0.75 to 0.88 [40]. Our correlation coefficient of 0.85 is in line with these findings. Most CPS tests also showed acceptable reliability, for example, ICC values of 0.88 and 0.97 were reported for the Processing Speed Test (PST) [41] and the computerized version of SDMT [18], respectively. The 95% CI on the mean ICC that we find ranges from 0.717 to 0.945. This is thus also similar to that reported in prior work.

To our knowledge, there are 2 other smartphone-based SDMT apps for MS that should be mentioned as alternative solutions to monitor CPS. One is MSCopilot [24], which contains a digital SDMT variant and 3 other digital variants of tests in the MSFC. However, results were only reported for the combined digital MSFC assessment in comparison to MSFC z-scores. Data on the validity and reliability of their digital SDMT variants specifically have never been published to our knowledge. FLOODLIGHT [25] is another smartphone monitoring app for persons with MS, which was developed by Roche. At a poster presented at the European Committee for Treatment and Research in Multiple Sclerosis in 2018, Montalban et al [25] reported a Spearman’s correlation of 0.615 between the FLOODLIGHT smartphone-based SDMT and the conventional in-clinic outcome measure (oral SDMT). This is considerably lower than our correlation coefficients (ie, Pearson *r*=0.85).

Well-validated digital assessment devices for CPS screening include the PST and the recently introduced Multiple Screener tool, which contains a digital SDMT for which an ICC of 0.79 between digital and paper-and-pencil-based assessment was reported [42]. Rudick et al [23] reported a Pearson correlation coefficient of 0.80 between the PST and analogous technician tests and concordance correlation coefficients to quantify test-retest reproducibility of 0.853 for the technician and 0.867 for persons with MS. Our results are similar to theirs. However, both the PST and Multiple Screener are only available for iPads and are intended to be used for monitoring CPS in a more controlled setting (in the clinic), whereas the MS sherpa app is intended to be used for home monitoring. The importance of personalization and customization of smartphone apps for persons with MS has been noted in other studies [43,44]. It is therefore recommended that the needs and context of the individual with MS are taken into account in the design of apps for persons with MS.

To our knowledge, this is the first study in which a smartphone variant of the SDMT that can be done unsupervised was cross-platform validated. We obtained valuable novel insights into frequent home monitoring with the SDMT such as the observation that the practice effect was only nontrivial between the first and second sSDMT (with 10 assessments scheduled and approximately 3 days in between each assessment) but also about the cumulative practice effects that are involved. Furthermore, we believe the qualitative insights obtained from patient interviews on the needs and wishes of smartphone-based home monitoring using sSDMT can inspire developers, caregivers, and researchers for future developments.

### Conclusion

This study shows the construct validity of the sSDMT since the ICCs(3,1) between the SDMT and the sSDMT for persons with MS and HC subjects were 0.78 and 0.85, respectively. The sSDMT does have very good test-retest reliability because only the first test-retest of the persons with MS and the HC matched group yielded an ICC(A,1) smaller than 0.80. All the other ICCs were higher than 0.80, both for persons with MS and for HC subjects. We conclude that the sSDMT has the potential to be used as a tool to monitor CPS in persons with MS, both in clinical studies and in clinical practice.

## Appendix

Multimedia Appendix 1 Number of correct answers on the SDMT and the sSDMT and the agreement between the two methods, for the 37 validation assessments that were done with the SDMT.

Multimedia Appendix 2 Number of correct answers on the vSDMT and the sSDMT and the agreement between the two methods, for the 54 validation assessments that were done with the vSDMT.

Multimedia Appendix 3 Number of correct answers on the sSDMT on 10 occasions (labeled 1 to 10) for the persons with multiple sclerosis and the healthy controls in the matched group.

Multimedia Appendix 4 Number of correct answers on the sSDMT on three occasions (labeled 1, 2 and 3) for the healthy control normative group.

## Abbreviations

2MWT: 2-minute walking test
BICAMS: Brief International Cognitive Assessment for Multiple Sclerosis
CPS: cognitive processing speed
EDSS: Expanded Disability Status Scale
HC: healthy control
ICC: intraclass correlation coefficient
MS: multiple sclerosis
MSFC: Multiple Sclerosis Functional Composite
NMSF: Nationaal MS Fonds
PASAT: Paced Auditory Serial Addition Test
s2MWT: smartphone 2-minute walking test
SDMT: Symbol Digit Modalities Test
sSDMT: smartphone variant of Symbol Digit Modalities Test
sWBT: smartphone walking balance test
TUG: Timed Up and Go
vSDMT: variant of the Symbol Digit Modalities Test
