# Supplementary material for: Symbol Digit Modalities Test Variant in a Smartphone App for Persons With Multiple Sclerosis: Validation Study
Source: JMIR Mhealth Uhealth. 2020 Oct 5;8(10):e18160. doi: 10.2196/18160 (PMC7573704; doi:10.2196/18160)
Supplement: Multimedia Appendix 1 [file mhealth_v8i10e18160_app1.docx]

**Multimedia Appendix 1.** Number of correct answers on the SDMT and the sSDMT and the agreement between the two methods, for the 37 validation assessments that were done with the SDMT. The administration codes are listed in the "code" column. The codes of persons with multiple sclerosis start with the letter A, those of healthy controls in the matched group start with the letter B. SDMT: Symbol Digit Modalities Test; sSDMT: smartphone variant of Symbol Digit Modalities Test.

|  | **code** | **SDMT** | **sSDMT** | **mean** | **∆ score** | **∆/mean** |
| --- | --- | --- | --- | --- | --- | --- |
|  |  |  |  |  |  |  |
| 1 | A01-1 | 51 | 54 | 52.5 | 3 | 5.7% |
| 2 | A02-1 | 41 | 32 | 36.5 | -9 | -24.7% |
| 3 | A03-1 | 53 | 41 | 47 | -12 | -25.5% |
| 4 | A04-1 | 33 | 31 | 32 | -2 | -6.3% |
| 5 | A05-1 | 58 | 45 | 51.5 | -13 | -25.2% |
| 6 | A18-1 | 52 | 55 | 53.5 | 3 | 5.6% |
| 7 | A18-2 | 74 | 63 | 68.5 | -11 | -16.1% |
| 8 | A24-1 | 54 | 42 | 48 | -12 | -25.0% |
| 9 | A25-1 | 49 | 44 | 46.5 | -5 | -10.8% |
| 10 | A26-1 | 45 | 43 | 44 | -2 | -4.5% |
| 11 | A26-2 | 54 | 47 | 50.5 | -7 | -13.9% |
| 12 | A28-1 | 60 | 57 | 58.5 | -3 | -5.1% |
| 13 | A28-2 | 58 | 67 | 62.5 | 9 | 14.4% |
| 14 | B03-1 | 73 | 58 | 65.5 | -15 | -22.9% |
| 15 | B03-2 | 84 | 74 | 79 | -10 | -12.7% |
| 16 | B09-1 | 52 | 46 | 49 | -6 | -12.2% |
| 17 | B09-2 | 54 | 59 | 56.5 | 5 | 8.8% |
| 18 | B13-1 | 36 | 33 | 34.5 | -3 | -8.7% |
| 19 | B15-1 | 50 | 44 | 47 | -6 | -12.8% |
| 20 | B15-2 | 47 | 45 | 46 | -2 | -4.3% |
| 21 | B16-1 | 52 | 42 | 47 | -10 | -21.3% |
| 22 | B16-2 | 52 | 50 | 51 | -2 | -3.9% |
| 23 | B19-1 | 47 | 39 | 43 | -8 | -18.6% |
| 24 | B20-1 | 69 | 60 | 64.5 | -9 | -14.0% |
| 25 | B20-2 | 75 | 62 | 68.5 | -13 | -19.0% |
| 26 | B21-1 | 54 | 52 | 53 | -2 | -3.8% |
| 27 | B21-2 | 55 | 56 | 55.5 | 1 | 1.8% |
| 28 | B22-1 | 62 | 54 | 58 | -8 | -13.8% |
| 29 | B22-2 | 70 | 60 | 65 | -10 | -15.4% |
| 30 | B23-1 | 76 | 60 | 68 | -16 | -23.5% |
| 31 | B23-2 | 71 | 59 | 65 | -12 | -18.5% |
| 32 | B24-1 | 63 | 48 | 55.5 | -15 | -27.0% |
| 33 | B24-2 | 66 | 52 | 59 | -14 | -23.7% |
| 34 | B27-1 | 62 | 47 | 54.5 | -15 | -27.5% |
| 35 | B27-2 | 59 | 56 | 57.5 | -3 | -5.2% |
| 36 | B28-1 | 69 | 60 | 64.5 | -9 | -14.0% |
| 37 | B28-2 | 70 | 68 | 69 | -2 | -2.9% |
|  |  |  |  |  |  |  |
| **mean** | |  |  |  | -6.62 | -12.06% |
| **standard deviation (s)** | | | |  | 6.13 | 10.68% |
