# Supplementary material for: Symbol Digit Modalities Test Variant in a Smartphone App for Persons With Multiple Sclerosis: Validation Study
Source: JMIR Mhealth Uhealth. 2020 Oct 5;8(10):e18160. doi: 10.2196/18160 (PMC7573704; doi:10.2196/18160)
Supplement: Multimedia Appendix 3 [file mhealth_v8i10e18160_app3.docx]

**Multimedia Appendix 3.** Number of correct answers on the sSDMT on 10 occasions (labeled 1 to 10) for the persons with multiple sclerosis and the healthy controls in the matched group. An asterisk (*) indicates that the test score was below 20. An asterisk after the test score (i.e. 30*) indicates that the test could not be not used to determine the test-retest reliability, because the test afterwards was not done or removed from the data. An em dash at the end of the series (—) indicates that the test was not done. The administration codes of persons with multiple sclerosis start with the letter A, those of healthy controls in the matched group start with the letter B. sSDMT: smartphone variant of Symbol Digit Modalities Test.

| **code** | **1** | **2** | **3** | **4** | **5** | **6** | **7** | **8** | **9** | **10** |
| --- | --- | --- | --- | --- | --- | --- | --- | --- | --- | --- |
|  |  |  |  |  |  |  |  |  |  |  |
| A01 | 54 | 44 | 51 | 47 | 51 | 58 | 51 | 52 | 61 | 56 |
| A02 | 32 | 34 | 36 | 31 | * | 41 | 40 | 37 | 39 | 36 |
| A03 | 41 | 42 | 43 | 45 | 42 | 47 | 51 | 48 | 48 | 50 |
| A04 | 31* | * | 36 | 35 | 34 | 36 | 36 | 42 | 40 | 41 |
| A05 | 45 | 48 | 51 | 47 | 49 | 47 | 49 | 49 | 51 | 44 |
| A06 | 42 | 45 | 44 | 43 | 46 | 47 | 47 | 46 | 41 | 45 |
| A07 | 41 | 44 | 46 | 48 | 46 | 48 | 46 | 49 | 45 | 51 |
| A08 | 45 | 54 | 49 | 54 | 59 | 55 | 60 | 54 | 58 | 55 |
| A09 | 37 | 42 | 42 | 37 | 43 | 43 | 44 | 45 | 41 | — |
| A10 | 44 | 52 | 49 | 49 | 59 | 45 | 57 | 57 | — | — |
| A11 | 52 | 51 | 56 | 60 | 54 | 55 | 53 | 59 | 58 | 67 |
| A12 | 44 | 56 | 52 | 55 | 47 | 53 | 57 | 53 | 58 | 55 |
| A13 | 25* | * | 23 | 32 | 31 | 32 | 29 | 27 | 33 | 38 |
| A14 | 41 | 40 | 37 | 44 | 39 | 41 | 39 | 44 | 41 | 38 |
| A15 | 33 | 37 | 39 | 38 | 45 | 41 | 43 | 44 | 43 | 43 |
| A16 | 47 | 51 | 51 | 50 | 52 | 58 | 53 | 55 | 55 | — |
| A17 | 48 | 51 | 51 | 45 | 46 | 62 | 52 | 49 | 57 | 54 |
| A18 | 55 | 55 | 60 | 57 | 61 | 65 | 63 | 57 | 62 | 63 |
| A19 | 50 | 55 | 54 | 54 | 57 | 56 | 61 | 61 | 61 | 65 |
| A20 | 52 | 60 | 61 | 62 | 60 | 68 | 66 | 63 | 67 | 65 |
| A21 | 57 | 58 | 61 | 56 | 63 | 59 | 66 | 66 | 64 | 78 |
| A22 | 33 | 34 | 36 | 40 | 39 | 43 | 45 | 39 | 43 | 44 |
| A24 | 42 | 51 | 51 | 47 | 52 | 47 | 49 | 46 | — | — |
| A25 | 44 | 47 | 46 | 44 | 44 | 46 | 42 | — | — | — |
| A26 | 43 | 47 | 46 | 50 | 46 | 49 | 46 | 48 | 46 | 48 |
| A28 | 57 | 68 | 66 | 60 | 68 | 63 | 70 | 69 | 65 | 69 |
| B01 | 55 | 53 | 57 | 55 | 57 | 65 | 62 | 64 | 62 | 68 |
| B03 | 58 | 65 | 69 | 71 | 74 | 72 | 72 | 72 | 74 | 72 |
| B05 | 52 | 62 | 60 | 54 | 63 | — | — | — | — | — |
| B07 | 52 | 65 | 61 | 61 | 57 | 64 | 62 | 66 | — | — |
| B08 | 36 | 42 | 44 | 49 | — | — | — | — | — | — |
| B09 | 46 | 44 | 44 | 49 | 50 | 47 | 53 | 47 | 51 | 45 |
| B11 | 56 | 54 | 53 | 57 | 53 | 59 | 53 | 58 | 62 | — |
| B12 | 49 | 51 | 55 | 60 | 58 | 63 | 65 | 55 | — | — |
| B13 | 33 | 36 | 39 | 43 | 44 | 41 | 40 | 44 | 44 | 42 |
| B15 | 44 | 37 | 48 | 48 | 49 | 45 | — | — | — | — |
| B16 | 42 | 43 | 48 | 48 | 46 | 48 | 44 | 51 | 50 | — |
| B17 | 46 | 58 | 56 | 53 | 65 | 56 | 62 | 61 | 60 | 64 |
| B18 | 42 | 47 | 39 | 46 | 45 | 46 | 49 | 50 | 47 | 55 |
| B19 | 39 | 48 | 50 | 45 | 55 | 50 | 53 | 53 | 51 | 51 |
| B20 | 60 | 56 | 63 | 62 | — | — | — | — | — | — |
| B21 | 52 | 57 | 54 | 51 | 54 | 46 | 50 | 54 | 55 | 55 |
| B22 | 54 | 54 | 54 | 57 | 60 | 60 | 58 | 60 | 60 | 60 |
| B23 | 60 | 59 | 58 | 62 | 56 | 59 | — | — | — | — |
| B24 | 48 | 47 | 53 | 54 | 50 | 51 | 50 | 54 | 52 | — |
| B25 | 60* | — | — | — | — | — | — | — | — | — |
| B27 | 47 | 56 | 53 | 55 | 56 | 57 | 61 | 56 | — | — |
| B28 | 60 | 62 | 64 | 67 | 68 | — | — | — | — | — |
