# Supplementary material for: Symbol Digit Modalities Test Variant in a Smartphone App for Persons With Multiple Sclerosis: Validation Study
Source: JMIR Mhealth Uhealth. 2020 Oct 5;8(10):e18160. doi: 10.2196/18160 (PMC7573704; doi:10.2196/18160)
Supplement: Multimedia Appendix 4 [file mhealth_v8i10e18160_app4.docx]

**Multimedia Appendix 4.** Number of correct answers on the sSDMT on three occasions (labeled 1, 2 and 3) for the healthy control normative group, whose administration codes (listed in the "code" column) start with the letter C. sSDMT: smartphone variant of Symbol Digit Modalities Test.

| **code** | **sSDMT-1** | **sSDMT-2** | **sSDMT-3** |
| --- | --- | --- | --- |
|  |  |  |  |
| C01 | 64 | 67 | 71 |
| C02 | 48 | 48 | 54 |
| C03 | 59 | 66 | 66 |
| C04 | 69 | 58 | 61 |
| C05 | 60 | 60 | 61 |
| C06 | 42 | 45 | 55 |
| C07 | 51 | 54 | 43 |
| C08 | 71 | 66 | 67 |
| C09 | 40 | 42 | 39 |
| C10 | 48 | 46 | 49 |
| C11 | 66 | 78 | 70 |
| C12 | 52 | 60 | 62 |
| C13 | 61 | 65 | 59 |
| C14 | 44 | 44 | 52 |
| C15 | 38 | 50 | 46 |
| C16 | 49 | 55 | 62 |
| C17 | 58 | 58 | 59 |
| C18 | 37 | 36 | 41 |
| C19 | 41 | 41 | 43 |
| C20 | 45 | 49 | 53 |
| C21 | 64 | 62 | 71 |
| C22 | 73 | 74 | 77 |
| C23 | 56 | 57 | 64 |
| C24 | 42 | 40 | 39 |
| C25 | 50 | 53 | 48 |
| C26 | 45 | 44 | 47 |
| C27 | 43 | 45 | 42 |
| C28 | 51 | 49 | 59 |
| C29 | 50 | 54 | 38 |
| C30 | 64 | 65 | 72 |
| C31 | 48 | 52 | 55 |
| C32 | 52 | 57 | 58 |
| C33 | 58 | 56 | 56 |
| C34 | 52 | 56 | 58 |
| C35 | 49 | 52 | 47 |
| C36 | 51 | 52 | 55 |
| C37 | 50 | 51 | 55 |
| C38 | 52 | 54 | 55 |
| C39 | 52 | 48 | 46 |
| C40 | 61 | 66 | 71 |
| C41 | 47 | 51 | 54 |
| C42 | 62 | 68 | 66 |
| C43 | 49 | 53 | 54 |
| C44 | 60 | 63 | 71 |
| C45 | 63 | 69 | 67 |
| C46 | 58 | 61 | 58 |
| C47 | 61 | 65 | 69 |
| C48 | 52 | 49 | 50 |
| C49 | 48 | 50 | 49 |
| C50 | 61 | 56 | 54 |
| C51 | 62 | 62 | 67 |
| C52 | 76 | 69 | 72 |
| C53 | 45 | 53 | 52 |
| C54 | 54 | 54 | 51 |
| C55 | 59 | 67 | 61 |
| C56 | 53 | 55 | 58 |
| C57 | 55 | 55 | 56 |
| C58 | 42 | 42 | 45 |
